# Spontaneous Pulmonary Hematoma: Case Report of a Giant Post-COVID-19 Hematoma and Literature Review

**DOI:** 10.3390/healthcare11040527

**Published:** 2023-02-10

**Authors:** Cornel Adrian Petreanu, Silviu Vlăsceanu, Dragoș Zaharia, Daniela Jipa, Horațiu Moldovan, Daniela Gheorghiță, Luminița Iliuță, Bogdan Rădulescu, Ioana Anca Bădărău, Cornel Florentin Savu

**Affiliations:** 1 Department of Thoracic Surgery, “Marius Nasta” National Institute of Pneumology, 050152 Bucharest, Romania; 2 Thoracic Surgery Clinic I, “Carol Davila” University of Medicine and Pharmacy, 030167 Bucharest, Romania; 3 Department of Physiology, “Carol Davila” University of Medicine and Pharmacy, 030167 Bucharest, Romania; 4 Department of Pneumology, “Marius Nasta” National Institute of Pneumology, 050152 Bucharest, Romania; 5 Pneumology Clinic, “Carol Davila” University of Medicine and Pharmacy, 030167 Bucharest, Romania; 6 Department of Cardiovascular Surgery, Faculty of Medicine, “Carol Davila” University of Medicine and Pharmacy, 050474 Bucharest, Romania; 7 Department of Cardiovascular Surgery, Clinical Emergency Hospital Bucharest, 014461 Bucharest, Romania; 8 Academy of Romanian Scientists, 050045 Bucharest, Romania; 9 Faculty of Materials Science and Engineering, Politehnica University of Bucharest, 060042 Bucharest, Romania; 10 Department of Medical Informatics and Biostatistics, Faculty of Medicine, “Carol Davila” University of Medicine and Pharmacy, 050474 Bucharest, Romania; 11 Department of Cardiovascular Surgery, Prof. Dr. C.C. Iliescu Emergency Institute for Cardiovascular Diseases, 022322 Bucharest, Romania

**Keywords:** spontaneous pulmonary hematoma, antiplatelet treatment, COVID-19 infection (coronavirus disease caused by the SARS-CoV-2 virus), emphysema bullae

## Abstract

Pulmonary hematomas are a rare pathology. Although they are usually reported post-traumatically, there are also spontaneous forms in pulmonary pathologies or during drug therapy. In these spontaneous entities, primitive forms are rarely described, although the contributory local pulmonary pathological terrain or a specific associated medication has not yet been identified. We present the case of a patient who developed a giant pulmonary hematoma that appeared spontaneously during recovery from COVID-19 infection. It appeared in one of the two bullae-like cystic lung lesions developed during secondary COVID-19 infection. The clinical impact was major, with hypotension and anemia being observed, requiring hemodynamic support and the adjustment of drug therapy. The clinical course was favorable, with a quasi-complete resolution of both the hematoma and a second cystic lesion being observable at 8 months by pulmonary remodeling. Spontaneous pulmonary hematomas may constitute a pathological entity associated with a post-COVID-19 remodeling process of the lung and the related anticoagulant treatment, which should be recognized, especially in the actual COVID-19 pandemic or in the widespread use of anticoagulant treatment. Conservative treatment is the method of choice, even in giant lung forms.

## 1. Introduction

Pulmonary hematomas are rarely reported anatomopathological forms, and truly spontaneous ones are exceptional. They are usually associated with anticoagulant treatments, but there are also systemic or local pulmonary pathological conditions. Most pulmonary hematomas, spontaneous or not, show a favorable clinical course under conservative treatment, with slow resorption; hence the Anglo-Saxon name “vanishing tumor” [1].

Post-traumatic or secondary hematomas may result from penetrating or non-penetrating injuries and we can identify the trigger and the moment of the appearance of hematomas. The extent of the associated vascular or bronchial lesions, and the lack of spontaneous resorption under conservative treatment or superinfection determine the need for surgical treatment.

Spontaneous forms, however, occur without a known specific trigger in some pulmonary diseases or during anticoagulant treatment, and the gold standard is conservative treatment. Real primitive forms are rarely reported, where a pathological terrain is identified in the absence of anticoagulant treatment [2].

COVID-19 infection (Coronavirus disease caused by the SARS-CoV-2 virus) triggers severe local anatomopathological changes with specific pneumonic lesions, honeycomb lesions, cystic lesions, infarction, and thromboembolisms [3,4]. The processes are secondary to local microcirculatory disturbances, with vasculitis. Thus, necrotic lesions and local microhemorrhage may occur, which may lead to intraparenchymal hematoma if the wall of cystic lesions is affected. These parenchymal reshaping and remodeling phenomena occur during convalescence and last for months.

## 2. Case Report

**Figure 1:**
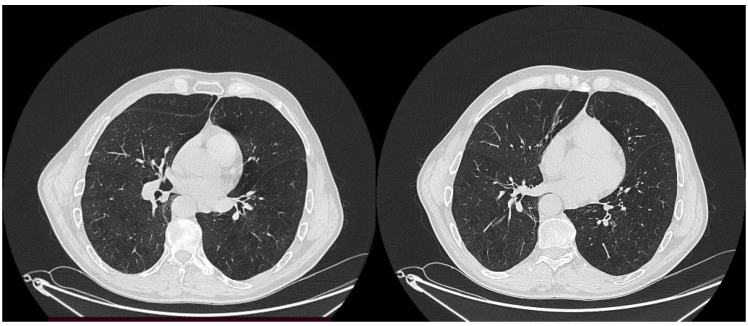
CT scan (2019) before COVID-19 infection, for evaluation of chronic respiratory insufficiency—diffuse lung emphysema.

We present the case of a 56-year-old patient, a heavy smoker (over 40 packs a year), with chronic respiratory insufficiency, without a history of right chest trauma, during the recuperation period after 17 days COVID-19 infection confirmation. He was known to have chronic respiratory insufficiency by our service of pneumology, with spontaneous oxygen saturation of 90–92% with ambient air, and moderate dyspnea for effort. His physical examination showed decreased lung sounds in both lung fields without wheezes or crackles. There was no palpable lymph node enlargement. Biological laboratory studies, including a platelet count and coagulation profiles, yielded normal results. Pulmonary function tests showed a severe obstructive pattern; the forced expiratory volume in 1 second (FEV1) was 1.42 L (38% of predicted volume); the forced vital capacity (FVC) was 3,62 l; and the ratio of FEV1 to FVC was 39%. The diffusing capacity was 14.7 mL/mm Hg/min (58% of predictive capacity). Sputum analyses were negative for malignancy, bacterial infection and tuberculosis. CT exams (computer tomography exams) before hospitalization revealed an emphysematous lung, which is typical for heavy smoking, as presented in Figure 1.

**Figure 2:**
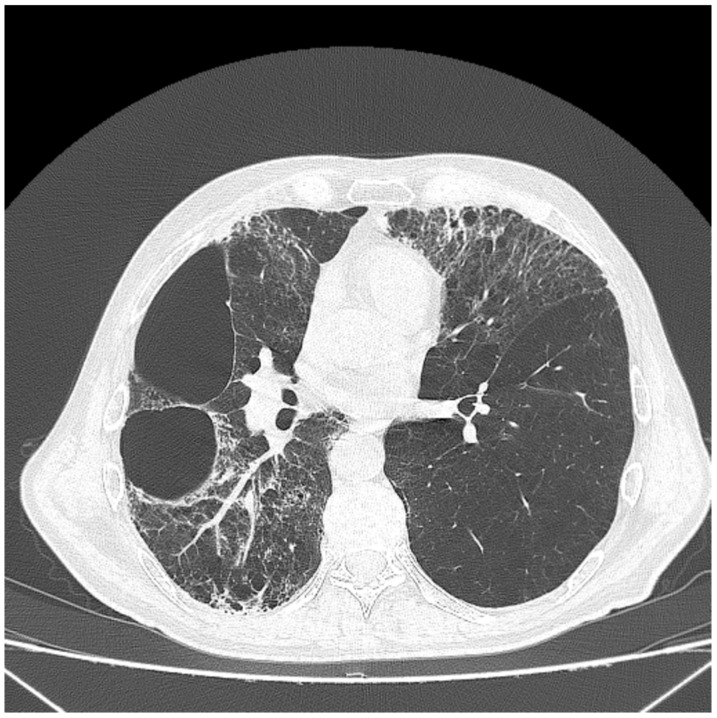
CT evaluation—2020—during acute viral infection indicated bilateral pneumonia, with ‘honeycomb’ lesions, which were confluent in the two big bullae on the right.

COVID-19 infection had a significant clinical impact, with aggravation of the dyspnea. The CT evaluation during acute viral infection indicated bilateral pneumonia, with ‘honeycomb’ lesions, especially on the right side. Some of these lesions were confluent in the big bullae lesion on the right side, presented in Figure 2. Following a COVID-19 infection, and after being treated with oxygen apport, corticoid anti-inflammatory, anticoagulant HMMG (heparin with low molecular height), and prophylactic antibiotic therapy, the patient was transferred to the rehabilitation service with good respiratory status in rest, but with oxygen required.

**Figure 3:**
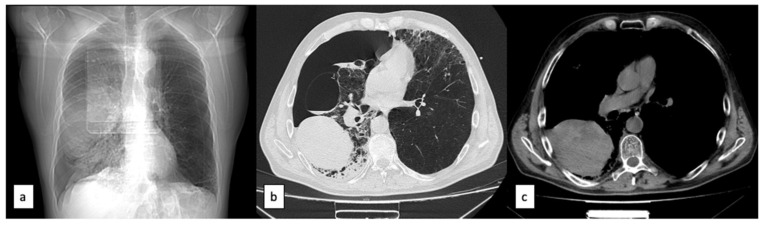
Scans indicate a right basal pneumothorax with opacification of bullous lesions: (**a**) X ray; (**b**) CT-lung window; (**c**) CT-chest window.

Suddenly, the patient complained of severe dyspnea during rest and right thoracic pain, with hypotension, tachycardia, and pallid face. The arterial blood gas test indicated severe hypoxia but the hemoglobin level also decreased (loss of 5 points). We performed a standard chest radiography, where a right basal pneumothorax with opacification of what could be bullous lesions was observed, as shown in Figure 3.

We identified a spontaneous right pneumothorax and a hemorrhage in one of the bullous lesions. The CT exam revealed the right pneumothorax and a large hematoma in one bullous lesion, measuring 11 cm/8 cm/14.2 cm.

The diagnosis was based on imaging and biological elements, correlated with the sudden drop in hemoglobin in the absence of any bleeding.

Following this, we decided on a conservative treatment. We immediately stopped the anticoagulant treatment, administered 1 blood unit, and performed pleural drainage for pneumothorax.

The clinical course was promising, with hemodynamic stability, as well as biological and clinical amelioration. The drainage was stopped after 3 days in the absence of air leaks. The anticoagulant treatment was reinstituted after 4 days. The patient was monitored for bacterial superinfection by blood analysis and an X-ray scan.

**Figure 4:**
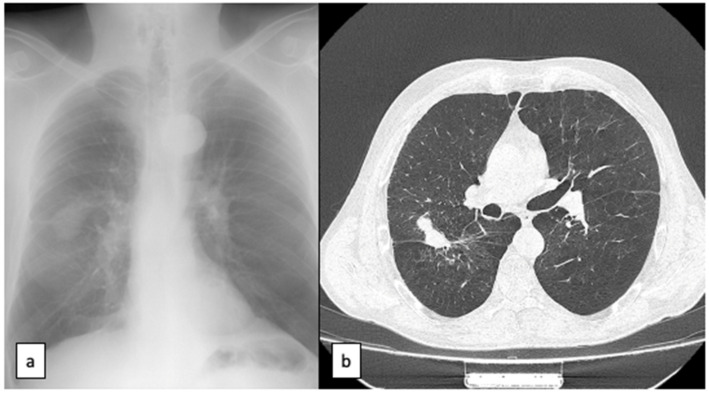
Control after 1 year (2022): (**a**) chest radiography; and (**b**) CT scan showing resorption of hematoma with chronicization of lesion, lack of other lung lesions—important remaniation.

After 2 weeks, the patient was discharged with corticoid and antibiotic medication and antiplatelet drugs, orally. The patient was followed for one year, clinically and radiologically. The CT scan after one year revealed a spontaneous resorption of the hematoma, transformed into a solitary nodule, slightly observed with a radiodensity of 40 HU (Hounsfield units). Additionally, the second parenchymal cystic lesion was reshaped and disappeared following remodeling, as presented in Figure 4.

## 3. Discussions

**Figure 5:**
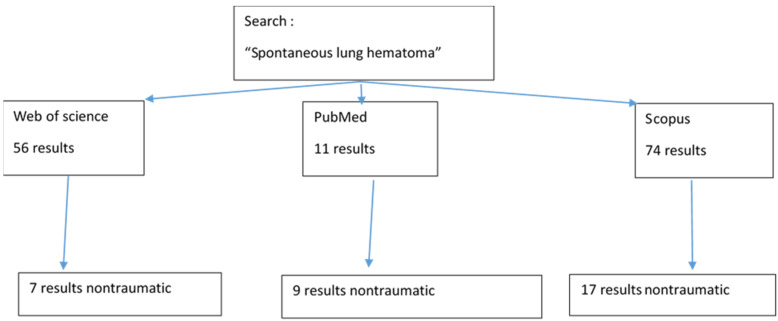
Literature research of “spontaneous lung hematoma” scheme.

In order to carry out the review, we performed a search on PubMed for the words “spontaneous lung hematoma” and found 11 results, of which only six were truly classified as spontaneous forms, the rest being excluded due to the post-traumatic context. The “spontaneous lung hematoma” analysis on the Web of Science identified 56 articles, from which we selected only seven clear cases. Figure 5 schematically represents the results obtained after literature research on Web of Science, PubMed and Scopus.

Additionally, a search of the words “lung hematoma” of the SCOPUS database identified 74 articles, of which 14 were accepted for research, the rest being post-traumatic or of other organs (Algorithm 1).

### 3.1. Etiopathogenesis

Most reports in the literature classify hematomas by mode of occurrence as spontaneous or post-traumatic. Thus, spontaneous pulmonary hematomas are usually associated only with anticoagulant therapy, but they have been described in patients with systemic or lung disease with this treatment [5,6].

Thus, those secondary to anticoagulant treatments are the most frequently reported in terms of spontaneous forms, probably arising from disorders of hemostasis, by disturbing the dynamic balance between the procoagulant components and the anticoagulant ones. Clinical cases have been reported with all types of anticoagulants, both oral and injectable, of the heparin type.

Considering that these hematomas are not related to the overdose of anticoagulant drugs, it is understandable that there is always one more favorable element.

Lee describes a truly primitive spontaneous pulmonary hematoma, without obvious underlying pulmonary pathology and without anticoagulant treatment. The diagnosis was made secondary to a thoracoscopic excision surgery [2]. However, if in the traumatic forms there are supposed to be ruptures of parenchymal vessels with secondary hemorrhage, phenomena arising from the deceleration or acceleration of areas with different density, in these spontaneous forms it is assumed that there are preexisting pathological, cystic areas, at the level of such phenomena as angionecrosis or erosion of the cystic wall. Alternatively, though the patients are not aware, a minor trauma to the chest may be the leading cause of pulmonary hematoma through blood vessel rupture, especially in the lung bases with much more mobility.

Similarly, there are reported cases that occurred spontaneously in patients treated with oral anticoagulants, without a previously evident underlying pathology [5,6]. In fact, in these cases there are certainly lesions undetected in advance, such as small emphysema bubbles or cysts.

The cases reported by Balala and Kent [7,8] of emphysematous lungs with cystic bullous lesions are explained on the same principle, in which, under anticoagulant treatment for the associated pathology, spontaneous hematomas appeared at the level of these pre-existing cavities. The incriminated mechanism was that of cystic parietal angiosclerosis, or their superinfection, with secondary hemorrhage. Similarly, Stephen Jay [9] presents a massive intrapulmonary hemorrhage related to infection in a bullous middle lobe lesion, resolved through drainage.

Usually, those appearing only under anticoagulant treatment are usually small on undetected lesions, with the larger forms being associated with known cystic or bullous lung lesions [10]. M. Mikubo et al. [11] report a spontaneous, nontraumatic posterior mediastinal hematoma in a patient on oral anticoagulant treatment, which required evacuation. In the same vein, Chakrwborty [12] describes the occurrence of pulmonary hematomas in patients treated with warfarin and heparin derivatives, within the uncontrolled anticoagulation [12,13,14,15,16]. Kok L.C. [17] signals the appearance of a first case of spontaneous pulmonary hematoma during heparin treatment, initiated for a myocardial infarction. Table 1 presents anticoagulant treatments reported in the occurrence of spontaneous pulmonary hematomas.

Additionally, the mechanism of disorder of the pro/anti-coagulant balance is also suspected in the spontaneous forms reported in thrombocytopenia [19] or in the forms encountered in patients who underwent thrombolytic treatment [20].

Usually, pulmonary hematomas are self-limiting due to the lower pressure regime in the pulmonary circulation, but if systemic vessels are involved, the events are precipitated, with immediate risk to life.

Although, in the reports found in the literature, spontaneous hematomas have a slight predominant location in the lower lobes, their location is decided by the position of the pre-existing lesions. In the traumatic ones, this dominance is more significant due to the greater exposure in this location [2].

Pulmonary sequestration is also a preformed cystic lesion, at the level of which spontaneous pulmonary hematomas can develop, secondary to superinfection and rupture of the parietal vessels. In the case of these pre-existing lesions, the phenomena can be dramatic due to the systemic pressure (and not pulmonary vessels), specific to their vascularization and due to the sometimes patent communication with tracheobronchial tree that favors tracheo-bronchial flooding. Wang [21] recently described such a case with a lethal course, secondary to hemorrhagia at the level of an intralobular sequestration, with hemothorax, pulmonary hematoma and major hemoptysis in the absence of a recent thoracopulmonary trauma.

The spontaneous mechanism may also be recorded in the report of a mediastinal hematoma associated with hemothorax by the rupture of a bronchial artery aneurysm fused to the peribronchial sheath [22,23]. Furthermore, Sersar et al. [24] report a case of pulmonary hematoma secondary to the rupture of an intraparenchymal vascular aneurysm.

In the same systemic pressure regime, spontaneous pulmonary hematoma was described in the context of a ruptured descending aorta aneurysm at the level of the left lower lobe [25]. The life-saving surgical intervention involved the resolution of the aneurysm and the practice of the left lower lobectomy to avoid possible complications due to the presence of a large hematoma superinfection. As a complication of the aortic prosthesis, the occurrence of left pulmonary hematomas was described in two patients with a history of thoracic aorta aneurysms [26].

Inflammatory phenomena with angionecrosis have also been implicated in the occurrence of pulmonary hematomas from superinfected bronchiectasis or in sepsis. Thus, in a sepsis with Staphylococcus aureus, Gerloni R. [27] reports the case of a 90-year-old woman who developed a left lower lobe peripheral hematoma that responded to conservative, antibiotic and anti-inflammatory treatment. The author mentions in the antecedents a chronic renal failure, with an effect on the coagulation balance and, in the imaging investigations, the presence of a diffuse emphysema, at the level of which the hematoma could have appeared spontaneously. In a similar case, Vlaovic [15] mentions the presence of double oral anticoagulant treatment for heart problems in a 75-year-old man. Similarly, Loperena A. [28] reports in a 75-year-old woman the appearance of a spontaneous pulmonary hematoma, probably due to superinfection and vascular rupture at the level of the bronchial submucosa of bronchiectasis of inferior lobes.

A special situation is described by Shindo, who performed a left upper lobectomy on a patient with repeated hemoptysis, with a spontaneous hematoma developed at the level of known bullous lesions [29]. A histopathological analysis of the resection piece revealed anaplastic carcinoma inside bulla of segment 1+2 with minimal invasion into the adhered parietal pleura, having a final stage T3N0M0. Survival was good over 7 years, with no signs of local recurrence. The retrospective analysis of 20 other identical cases identified the presence of these carcinomas associated with bubbles, as well as the cause of hemoptysis as a positive prognostic factor for earlier diagnosis.

Henoch-Schőnlein purpura is leukocytoclastic vasculitis, which rarely affects adults and occurs extremely rarely in the lungs. It is usually characterized by musculoskeletal, gastrointestinal, renal, cardiac, and ocular damage [30]. At the pulmonary level, the clinical course is unpredictable, being precipitated by the appearance of hemorrhagic infiltrates and pulmonary hematomas in such a patient under anticoagulant treatment. The mechanism is identical, disorder of the coagulation balance, at the level of the affected small–medium vascular walls.

Another pathology in which the occurrence of a spontaneous pulmonary hematoma was reported is diffuse amyloidosis with transthyretin, in which anticoagulant treatment was instituted for a cardiac arithmy [31]. The histopathological analysis of the nodule resection identified the presence of a lower lobe subpleural hematoma, without identifying ruptured vessels. There is, considering the mechanism of the hematoma formation, in addition to the hypermobility of the lower lobes, just above the diaphragm, abnormal amyloid deposition in the diffuse alveolar septal amyloidosis, mainly a mild emphysematous change of the lung and anticoagulant medication.

Systemic diseases such as Ehlers-Danlos syndrome were associated with the occurrence of spontaneous pulmonary hematomas in the absence of anticoagulant treatment, the suspected etiopathogenic mechanism being a vascular one, related to the fragility of the pleuro-pulmonary connective tissue specific to the syndrome [32]. The diagnosis was made by thoracoscopic exploration, and biochemical analysis of the cultured dermal fibroblasts and molecular biological examination revealed decreased production of type III collagen in the fibroblasts and COL3A1 mutation.

Sonokawa et al. [33] point out the occurrence of a spontaneous pulmonary hematoma in the absence of trauma or anticoagulant treatment in a patient with pulmonary diffuse ossification. Clinical presentation was spontaneous with hemoptysis sputum and right thoracic angina. Image evaluation revealed the presence of a right-sided pleurisy and of a large round mass in the lower long lobe, which were newly developed lesions. Pleural drainage revealed the presence of a hemothorax, whose abundant drainage required pulmonary resection. Analysis of the lesion revealed the present of diffuse ossifying lesions in the lobe, with granulation of tissue and an intrapulmonary hematoma. Given the extent of the hemorrhagic phenomena, it is possible that vascular erosion phenomena are involved in the hard (bony) lesions on the background of greater mobility in the juxta-diaphragmatic lower lobes.

Mizukami et al. [34] report the occurrence of visceral subpleural hematomas on the left diaphragmatic face, after a segmentectomy for long upper left lobe cancer. In the immediate postoperative period, they identified deep venous thrombosis, and introduced an antiplatelet treatment. The CT scan indicated cavities with air and liquid which required antibiotic therapy. Drainage was attempted under CT scan, but without success; surgery was then performed. Upon thoracoscopic inspection, we identified hematomas subpleural and practiced pleural excision with evacuation and a hemostatic suture. The author does not indicate if relaxing of the triangular ligament—a technical method used to release the lung to facilitate the total refill of pleural cavity after lung resection—was used. This surgical procedure is usually performed in the inferior part of the pleural cavity and may be an explication of a trauma on the diaphragmatic face of the lower lung lobe. Authors mentioned deep venous thrombosis of inferior legs, but these are source of lung infarction. If there were genuine hematomas linked to the anticoagulant treatment, their specific location on an area possibly involved in the surgical procedure remains in question.

A chronic expanding hematoma is an anatomopathological form, usually secondary to tuberculosis or with post-traumatic origins, which involves heterogeneous, thick-walled areas with calcifications. It increases due to self-perpetuation of the expansive process, via the irritating effect of blood or repeated bleedings [16,35,36,37].

In infants, the occurrence of spontaneous pulmonary hematomas associated with interstitial or alveolar hemorrhage is described, owing to the immaturity of tissues and vessels. They were described with a higher incidence in those not treated with surfactant, but more extensive in those treated. The incriminated mechanism is tissue and vascular rupture with interstitial hemorrhages and parenchymal hematomas in a lung lacking the surfactant element to keep the alveoli open. Likewise, alveolar hemorrhages appear to be apparently more extensive in those treated with surfactant [38].

Another unusual cause of pulmonary hematoma was the one identified by Sersar [24] in a 20-year-old man, who was known to have a vascular malformation—intraparenchymal aneurysm—and who spontaneously developed a intrapulmonary hematoma, with rapid growth. The surgical resection had a diagnostic and therapeutic role.

As in the presented case, there are reports of hemorrhagic phenomena associated with the COVID19 syndrome [39,40]. Thrombotic and embolic phenomena are demonstrated in all organs, as a common phenomenon in the cascade of the COVID syndrome. That is why prophylactic or even therapeutic anticoagulant medication was introduced. This is the only consensus, because it is missing regarding the type of anticoagulant, the dose or the duration of the treatment. Hemorrhagic intestinal parietal lesions with retroperitoneal or mesenteric extension are described more frequently, as well as other locations.

Thus, the study of Brogna et al. [41], similar to our case, report the spontaneous occurrence of hemothorax and subpleural lung hematoma in a patient with COVID-19. Thus, a CT scan 13 days after infection confirmed the diagnosis; the patient was under anticoagulant treatment of lower molecular weight heparin.

Additionally, Lozano et al. [42] reported two cases of spontaneous hematoma in 2021 in patients with COVID-19 infection under coagulant treatment, but these were secondary to pulmonary pneumonia infiltrates and were limited in size. No honeycomb lesions with a tendency of confluence were identified. In the present case, hemorrhage was more extensive due to the space in the emphysema bubbles, produced by the post-infection remissions, generating a giant intrapulmonary hematoma.

An interesting aspect is that these hematomas were not reported in the acute infectious phase, but in the recovery period, a sign that local remissions continue after this phase with the precipitation of complications.

Generally, spontaneous pulmonary hematomas have the tendency to be limited in size due to the fact that the bleeding occurs from pulmonary vascular systems with low pressure regimes, which limits expansion. The present case presents a giant hematoma due to bleeding from the wall of the giant bullae it filled.

Similar, Stephen Jay [9] presents a massive intrapulmonary hemorrhage unrelated to infection in a bullous middle lobe lesion, resolved through drainage. Moreover, spontaneous forms, besides the lack of a triggering element, do not have a specific location, an aspect specific to post-traumatic forms, which are mainly basally located [2].

Secondary traumatic forms have been described, especially in blunt trauma, but they can also be associated with penetrating ones and may have a different clinical picture and course from the spontaneous ones, frequently requiring surgical intervention [15,43,44,45,46,47,48,49,50,51,52].

Regarding spontaneous forms, the mechanism remains unclear. There are two pathophysiology theories during the anticoagulant treatment—they would occur through hemorrhages in pulmonary vascular lesions such as aneurysms, arteriovenous fistulas, or in those with emphysema through the appearance of parietal cystic changes secondary to infections, with the occurrence of angionecrosis and cystic wall erosion. A high level of C-reactive proteinemia suggests the infection precedes the cystic lesion wall necrosis [19].

Some authors suggest bleeding from pre-existing undetected pulmonary bullae or cysts, implying an infection in the wall with angionecrosis and bleeding sources. Regarding forms that appeared under anticoagulant treatment, local disorders of the procoagulant—anticoagulant balance or hemorrhage in microaneurysms—are suspected. Spontaneous forms associated with tumor-like lung lesions, especially sarcomatous, likely arise through neoplastic vascular erosion [29].

Microscopically, in the COVID-19 infection, damage of small vessels with thrombotic necrotizing capillary injury and septal capillary damage underlie the pulmonary lesions [41]. Thrombosis with perivascular inflammation is indicated by the appearance of an endothelial process and necrotizing or non-necrotizing vasculitis [42].

An inflammatory process appears in the vascular walls (vasculitis), with bleeding, ischemia, and necrosis, secondary to losing vascular integrity and luminal compromise. Thus, it was theorized that, in fact, COVID-19 infection represents a specific trigger for an abnormal immune response, underlying the occurrence of ANCA-associated vasculitis phenomena (circulating antineutrophil cytoplasmic antibodies) with diffuse alveolar hemorrhage, acute kidney injury, and purpuric manifestations as a subsequent complication, which is a post-acute COVID-19 syndrome.

The differential diagnosis for a single, discrete soft tissue density seen on chest radiography typically includes a primary lung neoplasm or solitary pulmonary metastasis, tuberculoma, aspergilloma, hamartoma, organizing pneumonia, Wegener’s granulomatosis, intrapulmonary lymph node, arteriovenous malformation and rheumatoid nodule. In patients with known bullous emphysema, this should also include infection or fluid within a bulla.

### 3.2. Imaging Diagnosis

Mainly, pulmonary hematomas are discovered via chest radiography, as well-circumscribed opacifications. When a pulmonary cystic mass is observed by clinicians by chest radiography, they should be aware of the possibility of a hematoma that is spontaneous or on a preexisting lesion, i.e., cystic.

Spontaneous hematomas are discovered unintentionally, unlike the post-traumatic ones, which are radiologically visible in the first 24–72 h from the event. The CT scan confirms the appearance of a fluid collection, of well-circumscribed opacity, with specific blood-fluid densities, being the gold standard in lung investigation. Olcaly et al. [53] raise the suspicion of hydatid cysts on CT examination, in the absence of IDR casoni in areas with epidemiological risk for parasitosis.

The diagnosis is an imaging one, associated with biological samples that indicate a decrease in hemoglobin, usually in the context of anticoagulant treatment. In a truly primitive form reported by Lee, thoracoscopic exploration was necessary to elucidate the diagnosis of pulmonary hematoma, but also CT-guided puncture is useful for diagnosis; subsequently, the therapeutic tactic can be decided [2].

In the acute phase, there is a lesion with increased density, and through the resorption process it decreases in size. Regarding the evolving densities, they may increase through the resorption and organization processes. Pozgain et al. [52] present a pleomorphic adenomatous primary tumor with CT imaging appearance resembling an intrapulmonary hematoma, but the diagnosis was suggested by aspiration cytology. Practically any collection or pulmonary tumor with similar densities in the CT scan should be included in the differential diagnosis. Komatsu et al. [54] present the limitations of the CT scan in evaluating a posterior mediastinal pulmonary hematoma, which is indistinguishable from a mediastinal tumor, discovered following trauma [55,56,57,58,59,60,61].

Non-traumatic forms are quite often mistaken for tumors because of their increased radiodensity, and differential diagnosis must also include malign lesions, especially in the resorption forms, when a spiculated appearance may occur as in the clinical case presented.

PET scan (positron emission tomography) was also invoked in the evaluation of pulmonary sclerosing pneumocytoma associated with pulmonary hematoma, indicating fixing capitation in the tumoral part and no capitation in hematoma area [36,37,62].

### 3.3. Clinical Course and Treatment

The clinical course is in most cases favorable, with slow resorption over months or years. Radiological monitoring is important. If it does not decrease in size in 4–6 weeks, needle evacuation or surgical excision is recommended, especially if an adjacent lesion is suspected. For children and young people, the monitoring period may be extended to 3–4 months [52,62]. From a pathophysiological point of view, the hematoma does not affect gas exchange and does not produce significant effects, but it presents a major risk of infection with abscess formation [62,63,64].

In large forms or associated with other pleural effusions, transthoracic drainage is necessary. In the case of superinfection with the appearance of an air–fluid level, antibiotic therapy is mandatory and should be administrated as a preventive treatment. Selected cases with stagnant or unfavorable course of abscessed hematomas or underlying associated lesions require regulated surgical resection [61,65].

The adaptation of anticoagulant treatment in the sense of temporary suspension and replacement of oral forms with more controllable injectable forms, the introduction of antibiotic therapy or the practice of plasmapheresis in the case of those associated with vasculitis are part of the routine therapeutic conduct.

The forms associated with the systemic circulation, diagnosed by antecedents of parenchymal arteriovenous malformations or pulmonary sequestration identified by imaging, with rapid growth and massive hemoptysis, require surgical exploration with a life-saving goal.

In the presented case, surgical resection was not an option due to chronic respiratory failure. Drainage was also not applied to favour a tamponade through lung parenchyma. Regulated surgical lung resection is reserved for traumatic forms with bronchial disruption and associated major bleeding [47,62,63,64,65,66].

## 4. Conclusions

Pulmonary hematomas represent a pathology not necessarily designed for surgery, with only selected cases requiring resection. Spontaneous forms are rarer, but challenging in diagnosis that the post-traumatic hematomas, and usually have a favorable clinical course under conservative treatment, even in giant forms.

Spontaneous pulmonary hematomas appear to be associated with long-term anticoagulant treatment, exceptionally without a pathological substrate and usually related to certain pulmonary pathologies and systemic diseases affecting the lung. The broad spectrum of reported pathologies associated with pulmonary hematoma practically suggests that the mechanisms are common ones of local infection and disorder of coagulation phenomena, with the appearance of angionecrosis and hemorrhages in the small circulation.

Spontaneous pulmonary hematomas are self-limited, due to the low blood pressure specific to the pulmonary circulation and due to the fact that hemorrhagic incidents usually occur at the level of limited parenchymal cavities, such as bullos emphysema. There is a predominant localization in the lower lobes that is more mobile peridiaphragmatically. Those that concern the systemic circulation have a louder, more dramatic clinical characteristic and tend to be more extensive.

COVID-19 infection facilitates—by vasculitic changes, especially when there is an associated anticoagulant treatment—the appearance of spontaneous pulmonary hematomas.

Post-COVID-19 remodeling process of the lung exceeds the contagious, acute infection period and can generate life-threatening injuries. Conservative treatment is prioritized in spontaneous uncomplicated infectious forms, with resorption even of giant cases.

The clinician must suspect any pulmonary nodule with evaluated imaging by computed tomography with liquid or mixed content, especially in patients with pulmonary diseases or systemic conditions, or with anticoagulant treatments.

## Figures and Tables

**Table 1:** Types of anticoagulant treatment associated with spontaneous pulmonary hematoma.

	Oral Anticoagulants	Heparine Derivates	Thrombolytic Agent
Goran et al. [18]		+	+
Riachy et al. [5]	+		
Hansen et al. [6]	+		
Kent et al. [7]	+		
Balala et al. [8]	+		
Ribeiro et al. [10]	+		
Chakraborty et al. [12]	+	+	
Kok et al. [17]		+	
Vlaovic and all [15]	+ +		

## Data Availability

The data presented in this study are available on reasonable request from the corresponding author.
